# Persistent asymptomatic eosinophilia after raw seafood exposure with normal IgE: a case report

**DOI:** 10.3389/fimmu.2026.1804368

**Published:** 2026-05-22

**Authors:** Wen He, Juzhang Li, Jianfeng Zhang, Qiaozhen Wu

**Affiliations:** Department of Respiratory and Critical Care Medicine, Suzhou Ninth Hospital Affiliated to Soochow University, Suzhou, China

**Keywords:** albendazole, asymptomatic eosinophilia, case report, eosinophilia, raw seafood exposure

## Abstract

Marine seafood–derived anisakid nematodes (e.g., *Anisakis* spp. and *Phocanema* spp. [*formerly Pseudoterranova* spp.]) may cause gastrointestinal and allergic manifestations. Here, we report an unusual case of persistent, asymptomatic peripheral eosinophilia following ingestion of raw or lightly marinated seafood, raising the possibility of subclinical antigenic stimulation. The patient remained entirely asymptomatic with normal total IgE levels, and parasite serology (including Angiostrongylus cantonensis IgG) was negative, yet developed marked eosinophilia. The patient had a childhood history of allergic diseases that fully resolved in adulthood, which may represent an immunologic background relevant to the unusual presentation of marked eosinophilia with persistently normal total IgE. Although the underlying mechanism could not be directly demonstrated, this pattern raises the possibility of a predominantly eosinophil-associated type 2 response without a prominent humoral allergic manifestation. The eosinophil count remained persistently elevated over several months and stayed markedly above the adult upper reference limit before treatment, while comprehensive evaluations for parasitic infection, infectious disease, and autoimmune conditions were negative. Bone marrow findings demonstrated reactive eosinophilic hyperplasia, and imaging studies were unremarkable. Following empiric albendazole therapy, the eosinophil count rapidly returned to normal. This temporal association was supportive of, but not diagnostic for, a parasite-related or antigen-mediated trigger in this otherwise unexplained case. This observation aligns with the latest UK Guideline for the Investigation and Management of Eosinophilia, which recommends considering empiric anthelmintic therapy in asymptomatic patients without an identifiable cause. This case suggests that in patients with otherwise unexplained asymptomatic eosinophilia, even a remote and limited history of consuming raw or lightly marinated seafood should not be overlooked during history taking, and that empiric anthelmintic therapy may offer practical diagnostic and therapeutic value in selected cases.

## 1 Introduction

Peripheral blood eosinophilia is a common clinical finding with diverse etiologies, including parasitic infections, allergic diseases, drug reactions, autoimmune disorders, and hematologic malignancies. When routine initial evaluations are unrevealing, such cases may be provisionally categorized as idiopathic and may require further assessment to exclude clonal or other secondary causes (1, 2). Marine seafood–derived anisakid nematodes are recognized pathogens capable of inducing eosinophilia. In humans, anisakid exposure may present with acute gastric symptoms, later intestinal involvement, allergic manifestations, or combinations of these features (3, 4). After ingestion by humans, anisakid larvae such as *Anisakis* spp. and *Phocanema* spp. generally cannot complete their life cycle and may die or become sequestered, leaving behind parasite-derived antigenic material (4–6). Such residual antigenic stimulation may contribute to eosinophilopoiesis, sometimes even in the absence of typical gastrointestinal symptoms or prominent IgE antibody responses (7, 8). The unique value of the present case lies in its unusual clinical presentation: persistent asymptomatic isolated eosinophilia, persistently normal total IgE, and a remote history of limited raw seafood exposure that became relevant only after a rigorous review of the patient’s medical records, exposure history, and dietary history. Rather than establishing a definitive immunologic mechanism, this case highlights the diagnostic challenge posed by otherwise unexplained eosinophilia and the potential clinical relevance of careful exposure-history assessment.

## 2 Case presentation

**Figure 1:**
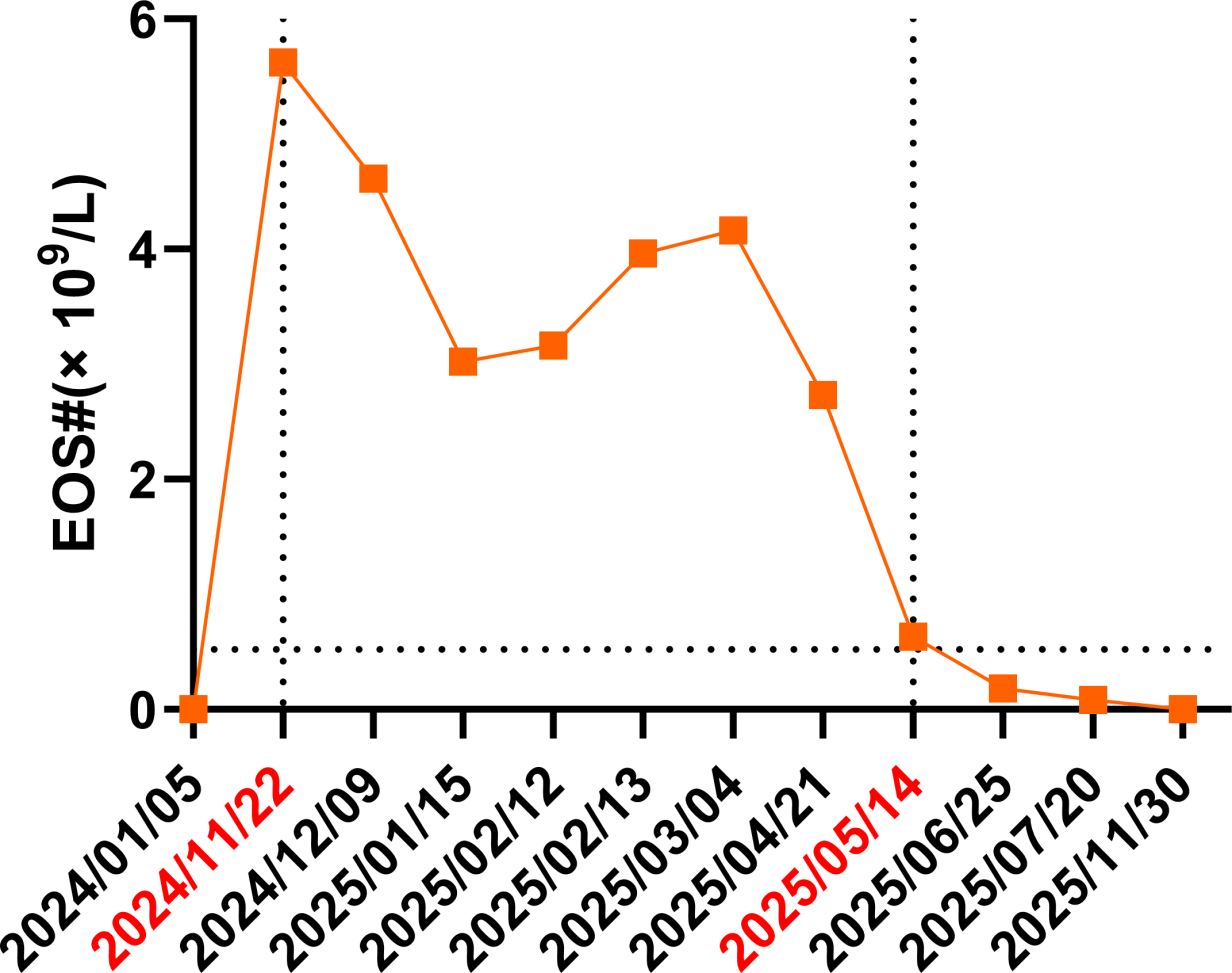
Serial absolute eosinophil count (EOS#) during the clinical course. The blue dotted line indicates the upper limit of normal eosinophil count (0.52 ×10^9^/L). The left vertical dotted line marks the date of the patient’s first abnormal eosinophil count detected on clinical testing (5.62 ×10^9^/L on November 22, 2024). The right vertical dotted line indicates the time point approximately one week after empiric albendazole therapy (initiated on May 8, 2025), when the eosinophil count declined to near-normal levels.

A 28-year-old man presented for evaluation after “persistent peripheral blood eosinophilia for more than five months” was incidentally identified during a routine health examination. His past medical history included childhood bronchial asthma and allergic rhinitis, for which he had been treated with inhaled Seretide (a combination of a β2-agonist and a corticosteroid). Notably, he also had a significant history of food allergies during childhood, particularly to fish, eel, mango, and peach. As he grew older, his asthma symptoms resolved completely, and at present he experiences only mild perioral itching after peach ingestion. He denied long-term medication use, chronic medical conditions, or a history of smoking. A routine health check in November 2024 revealed marked eosinophilia, with an absolute eosinophil count of 5.62×10^9^/L and an eosinophil percentage of 46.5%. According to the adult reference interval used by our clinical laboratory, the upper limit of the normal absolute eosinophil count is 0.52×10^9^/L. Notably, he remained entirely asymptomatic, with no abdominal pain, diarrhea, rash, pruritus, cough, wheezing, or fever, and chest/abdominal CT scans were unremarkable. Over the subsequent five months, repeated complete blood counts consistently showed sustained eosinophilia, with absolute eosinophil counts mostly ranging from 3.0–4.6×10^9^/L and remaining markedly above the adult upper reference limit before treatment. To clarify the etiology, a comprehensive differential diagnostic workup was performed. Serological testing for parasitic infections was negative, including assays for *Schistosoma*, *Clonorchis sinensis*, *Paragonimus westermani*, *Spirometra mansoni*, *Angiostrongylus cantonensis*, *Strongyloides stercoralis*, and *Trichinella spiralis*. Scrub typhus, dengue fever, epidemic hemorrhagic fever, and Brucella infection were also excluded. Three consecutive stool examinations detected no ova or parasites. Autoimmune markers (ANA, ANCA, and specific ANCA-associated antibodies) were within normal limits. Total IgE was within the normal reference range during the post-exposure diagnostic evaluation (138 IU/mL; laboratory reference range: <169 IU/mL). Serum IgG, IgA, IgM, and immunoglobulin subclasses were also within their respective reference ranges. Because no pre-exposure baseline IgE measurement was available, the normal IgE level should be interpreted as a post-exposure finding and does not exclude a prior transient IgE response or a predominantly eosinophil-associated immune reaction. In February 2025, bone marrow aspiration and biopsy were performed to rule out hematologic malignancy. Findings showed trilineage hematopoiesis with marked but morphologically normal eosinophilic proliferation. Flow cytometry detected no abnormal clones, and there was no evidence of marrow fibrosis. Overall, the results supported reactive eosinophilia. A rigorous review of the patient’s medical records, exposure history, and dietary history revealed a single intake of lightly marinated raw seafood—including shrimp, blood cockles, and marine fish—while he was in Guangzhou in April 2024. Although he experienced no gastrointestinal symptoms at the time or afterward, this history raised clinical suspicion of a possible seafood-related parasitic exposure, including exposure to marine nematodes such as *Anisakis* spp. or *Phocanema* spp. Given the exclusion of multiple other etiologies and the relevant exposure history, a parasite-related trigger was considered clinically plausible. To test this hypothesis, diagnostic anthelmintic therapy was initiated: albendazole 400 mg orally once daily for seven days beginning on May 8, 2025. The therapeutic response was rapid and marked: the eosinophil count decreased sharply from 2.73×10^9^/L before treatment to 0.63×10^9^/L, with a simultaneous normalization of the eosinophil percentage. Levels remained stable and normal throughout subsequent follow-up, with no recurrence. The swift and sustained hematologic response provided indirect but clinically meaningful support for a parasite-related trigger in this case, and suggested that empiric anthelmintic therapy may be informative in selected patients with otherwise unexplained eosinophilia. The longitudinal changes in peripheral eosinophil counts during the disease course are shown in **Figure 1**.

## 3 Discussion

This case illustrates an unusual clinical scenario of persistent asymptomatic isolated eosinophilia in which a remote history of limited raw seafood exposure became relevant only after extensive evaluation had failed to identify a clear cause. The diagnosis was not established through direct parasitological confirmation but was instead inferred from a coherent chain of indirect evidence. This chain began with the patient’s documented high-risk exposure to raw, marinated seafood prior to onset, followed by the core laboratory abnormality of persistent and marked peripheral eosinophilia. Notably, the absence of clinical symptoms and the lack of abnormalities on imaging supported a subclinical or low-burden parasite-related process rather than overt tissue-invasive disease. However, the present case does not allow a clear distinction between persistent low-level larval viability and a localized or sequestered parasite-related antigenic process that escaped detection by routine imaging and stool examinations. Endoscopic evaluation with gastroscopy or colonoscopy and mucosal biopsy was not performed because the patient remained entirely asymptomatic, reported no gastrointestinal complaints, and had no imaging abnormalities that would routinely prompt invasive gastrointestinal assessment in clinical practice. Nevertheless, we acknowledge that such investigations might have identified localized eosinophilic infiltration or granulomatous lesions and could have strengthened the etiologic inference. In contrast, higher parasite burden or more active tissue invasion would be more likely to produce overt gastrointestinal symptoms, imaging abnormalities, or granulomatous inflammatory lesions (5, 6, 9).

Serologically, the patient exhibited normal total IgE levels, and all parasite-specific assays available through routine clinical testing at our institution were negative. However, species-specific serology for *Anisakis* spp. and *Phocanema* spp. was not available in our hospital laboratory, and no routine reference laboratory service offering these tests was accessible during the diagnostic workup. Therefore, the attribution to marine nematodes remains inferential rather than directly confirmed. This may reflect, on the one hand, a relatively low or intermittent antigen load insufficient to elicit a robust humoral immune response (10); on the other hand, it may also reflect inter-individual variability in immune responsiveness and assay sensitivity. Subsequent bone marrow examination confirmed reactive eosinophilic hyperplasia, effectively arguing against a primary hematologic disorder. Importantly, the rapid and durable normalization of eosinophil counts after empiric albendazole requires cautious interpretation. If the process had been driven solely by residual non-viable parasite antigens, such a prompt hematologic response to a short course of an anthelmintic agent would be difficult to explain mechanistically. Therefore, the therapeutic response observed here may be more compatible with the presence of a persistent low-burden helminth-related trigger, including the possibility of ongoing larval viability or another biologically active parasite-associated component not detectable by routine imaging or stool examinations. At the same time, because direct parasitological confirmation was lacking, a localized antigen-driven immune reaction cannot be fully excluded. Taken together, these findings support the plausibility of a parasite-related process contributing to the eosinophilia observed in this case, while still falling short of definitive etiologic confirmation (11).

Of note, recent international evidence has also lent additional external support to the diagnostic and therapeutic reasoning applied here. The latest UK guideline on eosinophilia management (2) recommends that, in patients over 24 months old with unexplained eosinophilia—and after excluding specific contraindications such as high-grade *Loa loa* microfilaremia—empiric anthelmintic therapy (e.g., albendazole or albendazole combined with ivermectin) may be initiated without waiting for definitive parasitological confirmation, thereby achieving both diagnostic and therapeutic value. This strategy closely mirrors the logic used in the present case: when standard infectious and immunologic evaluations are negative but a relevant exposure history is present, empiric anthelmintic therapy may represent a pragmatic clinical step that is both feasible and potentially informative, and may help avoid unnecessary invasive investigations in appropriately selected patients. In the present case, the rapid normalization of eosinophil counts after albendazole may be more compatible with the existence of a still-removable parasite-related trigger than with a purely antigen-only state, although this interpretation remains inferential. Nevertheless, this observation is consistent with, and provides practical supportive evidence for, the “empiric therapy” approach recommended by the guideline.

**Figure 2:**
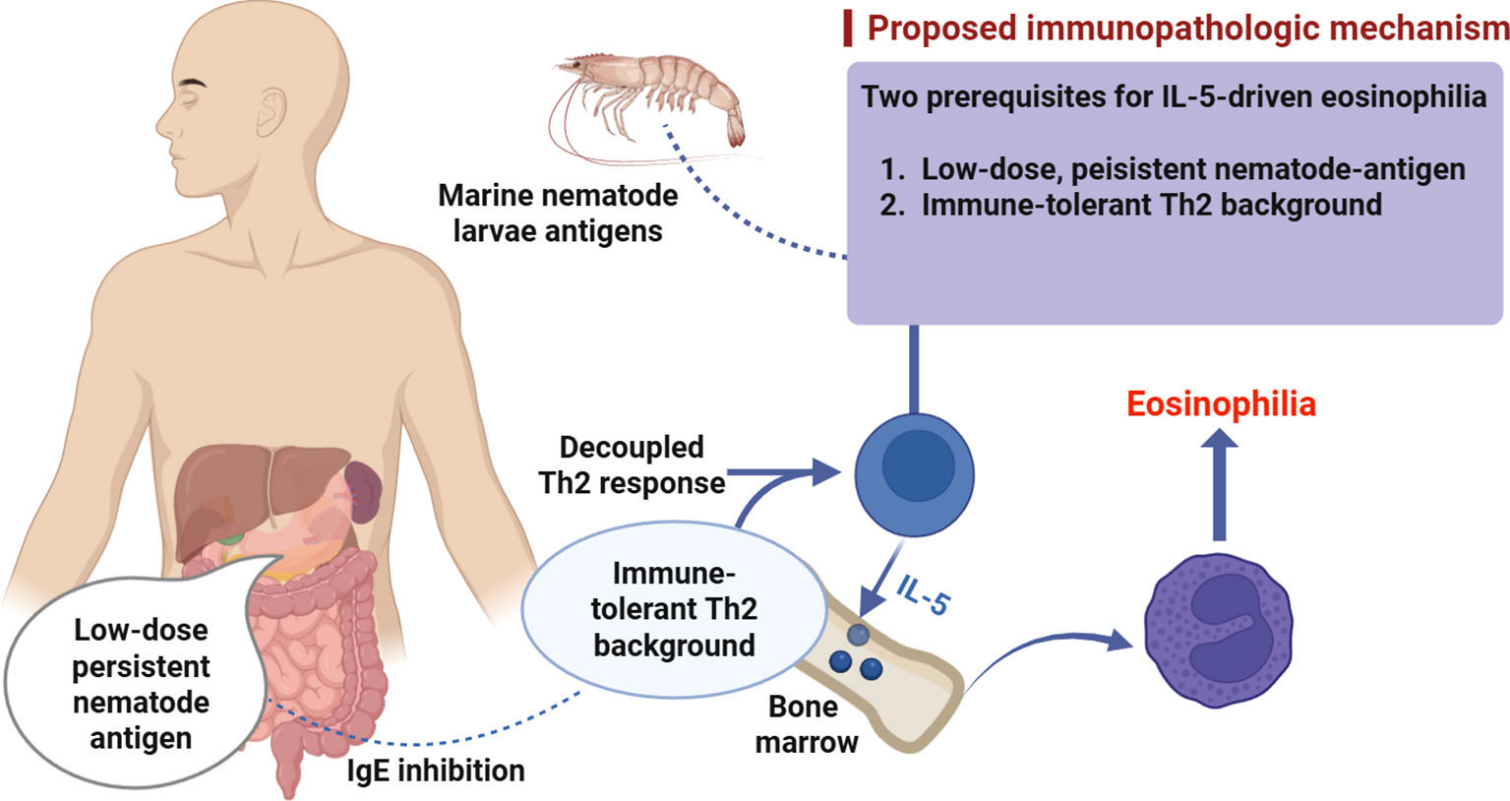
Hypothetical model for the possible immune basis of marked eosinophilia with normal total IgE after suspected seafood-related parasitic exposure.

The most notable immunologic feature of this case is marked eosinophilia in the presence of normal total IgE, a pattern that is compatible with a potentially “decoupled” type-2 response in which IL-5–axis involvement is plausible but inferred, as IL-5 activity was not directly measured. The patient’s childhood history of Th2-skewed diseases (asthma and allergic rhinitis), followed by spontaneous remission in adulthood, suggests that immune regulation may have evolved over time, potentially involving regulatory pathways such as Treg-associated mechanisms (12). We therefore propose—cautiously—that upon exposure to a low dose of nematode antigens, pre-existing type-2 immune memory might be re-engaged; meanwhile, regulatory signals (e.g., IL-10 and TGF-β) could attenuate B-cell IgE class switching and limit mast cell/basophil activation (13), thereby contributing to the absence of typical allergic symptoms and persistently normal IgE levels. In contrast, eosinophilopoietic signals within the type-2 pathway, including IL-5–related programs, may have remained relatively preserved, sustaining bone marrow eosinophilopoiesis and resulting in marked peripheral eosinophilia (7). This framework offers a plausible explanation for the observed phenotype—an eosinophil-dominant cellular type-2 response without overt humoral allergic manifestations—while remaining speculative in the absence of cytokine profiling or antigen-specific functional assays. The proposed immunopathogenic model is illustrated in **Figure 2**.

This case offers important implications for clinical practice. In the evaluation of otherwise unexplained eosinophilia, even a remote and limited history of consuming raw or lightly marinated seafood should not be overlooked during clinical history taking, particularly when parasitological serology is negative and classical symptoms are absent. After reasonable exclusion of other common causes, empiric anthelmintic therapy may be considered as a pragmatic step that may help reduce unnecessary invasive investigations, including bone marrow examination, in selected patients. Under these circumstances, the combination of “negative serology” and “marked eosinophilia” may not rule out a parasite-related process but instead point toward a subclinical parasite-related process, which could reflect either persistent low-burden viability or localized antigenic stimulation, rather than overt active infection.

Of course, this report has limitations. Most notably, parasite material or antigens could not be directly demonstrated in tissue, larval viability at the time of treatment could not be determined, and antigen-specific IL-5 responses could not be assessed using routine clinical assays. In addition, because gastroscopy or colonoscopy with mucosal biopsy was not performed, localized eosinophilic tissue infiltration or granulomatous lesions could not be fully excluded. Although the rapid and sustained normalization of eosinophil counts after albendazole therapy is clinically supportive, it should not be regarded as diagnostic evidence. Alternative explanations, including spontaneous resolution of eosinophilia over time or non-specific treatment-associated effects, cannot be completely excluded. Nevertheless, the persistence of marked eosinophilia for several months before treatment, the exclusion of multiple common infectious, allergic, autoimmune, and hematologic causes, the relevant seafood exposure history, and the temporal association with albendazole therapy together support a probable parasite-related or antigen-mediated trigger, while still falling short of definitive etiologic confirmation.

## 4 Conclusion

This case highlights a pragmatic clinical lesson in the evaluation of persistent asymptomatic eosinophilia: etiologic certainty may not always be achievable, but clinically relevant exposure history can still inform management. Even when the exposure occurred long before presentation and involved only a small amount of raw or lightly marinated seafood, such history may remain clinically relevant and should not be overlooked.

In regions where raw seafood consumption is common, seafood-related anisakid exposure should remain in the differential diagnosis of otherwise unexplained eosinophilia. After reasonable exclusion of other common causes, empiric anthelmintic therapy may be considered in selected patients as a practical diagnostic and therapeutic step, although the therapeutic response should be interpreted as supportive rather than definitive etiologic evidence.

## Data Availability

The original contributions presented in the study are included in the article/supplementary material. Further inquiries can be directed to the corresponding author.
